# Impacts of the COVID-19 pandemic on Chinese assisted reproductive technology institutions and human sperm banks: reflections in the post-pandemic era

**DOI:** 10.1186/s41043-023-00422-1

**Published:** 2023-08-18

**Authors:** Lun Wei, Jiakai Zhang, Xiaoling Deng, Chao Luo, Le Bo, Shasha Gao, Fei Qian, Shucheng Lu, Caiping Mao

**Affiliations:** 1 Reproductive Medicine Center, First Affiliated Hospital of Soochow University, No.899 Pinghai Road, Suzhou, 215006 Jiangsu China; 2 Centre for Chinese Urbanization Studies & Collaborative Innovation Center for New Urbanization and Social Governance of Universities in Jiangsu, Soochow University, No.50 Donghuan Road, Suzhou, 215021 Jiangsu China; 3 Suzhou High School Affiliated to Xi’an Jiaotong University, Suzhou, Jiangsu China

**Keywords:** Assisted reproductive technology, China, COVID-19, Human sperm bank, Post-pandemic era

## Abstract

**Objective:**

The COVID-19 pandemic has been the most serious public health emergency encountered in modern assisted reproductive technology (ART) development. In order to identify lessons learned, this study reviews the effect of the pandemic on ART institutions and human sperm banks in China, and summarizes the experiences and reflections of Chinese scholars post-pandemic era.

**Methods:**

This review is based on multiple consensus statements on the COVID-19 pandemic issued by Chinese experts as well as current national regulations and principles in ART institutions and human sperm banks to document the current situation of ART services in China, describe the impact of the pandemic on these services, and offer Chinese reflections on worrying issues in the post-pandemic era.

**Results:**

China reached one million ART cycles in 2016, and there are currently 540 ART medical institutions and 27 human sperm banks, with 540 licensed for AIH, 91 for AID, 415 for conventional IVF and ICSI and 85 for PGT. Of these, only 4 institutions carry out 10,000 cycles or more annually, and the proportion of institutions with less than 1,000 cycles has reached 66%, which means that a considerable number of ART institutions are still not saturated. As a consequence of the COVID-19 pandemic, 63.6% of ART providers and 95.5% of human sperm banks suspended operations. By the end of May 2020, China, as an early country affected by the pandemic achieved a national resumption rate of ART medical services of 99.2% and that of human sperm banks of 100.0%. Reports from the first and largest human sperm bank in China showed that qualification, semen concentration and sperm viability rates measured at primary screening have significantly decreased post-pandemic. Much like in other countries, Chinese experts developed a consensus on prevention and control measures during the pandemic. In principle, all ART activities should be suspended during acute phases of infection spread. Chinese scholars highlight that attention should be paid to young patients with fertility requirements during and after COVID-19, and emphasize the importance of fertility evaluation and clinical intervention. In addition, couples should be reminded that during ART treatment, disinfectants should not be used excessively to minimize risks of damaging the reproductive system, gametes and zygotes. At the same time, timely and reasonable guidance for tackling negative emotions from stress response is needed to provide reassurance and to avoid irrational fear and excessive stress. Seminal parameters should be re-examined 2 months after SARS-CoV-2 vaccination, and ART treatments recommenced if no abnormalities are detected.

**Conclusions:**

Given the growing frequency of outbreaks of global infectious diseases in recent years, ART institutions and human sperm banks should pay attention to improving their prevention and control capabilities. To a certain extent, decisions and measures adopted in China during COVID-19 pandemic are worthy of recognition and acceptance. Chinese scholars have discussed, proactively responded to and understand the key issues surrounding ART development during the pandemic with the aim of contributing to the substantial progress and healthy development of ART services in the world.

## Introduction

The COVID-19 outbreak in China in late 2019 and subsequent global pandemic have amounted to the most serious public health emergency encountered in modern ART development [1]. In the early stages of the COVID-19 pandemic, ASRM, ESHRE, BFS/ARCS, and CFAS all recommended immediate cessation of all reproductive health services except for emergencies [2, 3]. To date, more than 767 million cases have been diagnosed and more than 6.95 million deaths have occurred worldwide, and this number continues to grow [4]. Nowadays, COVID-19 prevention and control have been normalized, and countries have gradually resumed reproductive health services [5, 6].

As the first country to suffer from the COVID-19 pandemic, China was also one of the first countries in the world to control the epidemic domestically. In addition, China, with a population of 1.4 billion, is the most populous country in the world. Therefore, decisions and experiences from China can be of value globally, in ART institutions and human sperm banks. This review is based on multiple Chinese expert consensus statements on the COVID-19 pandemic as well as current national regulations and principles governing ART institutions and human sperm banks. Our aim is to report the current situation of ART services in China, describe the impact of the pandemic on and offer Chinese reflections on concerning issues in the post-pandemic era in order to contribute to the substantial progress and healthy development of ART services in the world.

## The current situation of ART in China

**Fig. 1:**
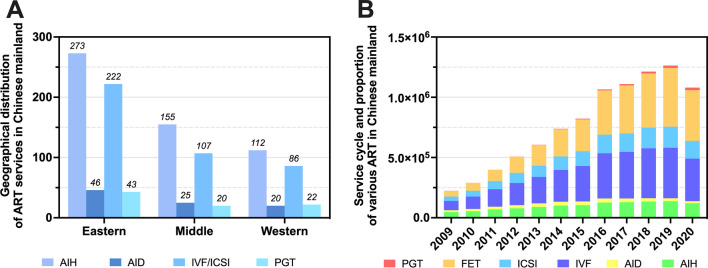
Statistics on the Current Situation of ART medical services in China. **a** Geographical distribution of ART services in Chinese mainland; **b** Service cycle and proportion of various ART in Chinese mainland

According to data published at the CSRM Annual Meeting in August 2022 [7], the proportion of ART live births has increased year by year. From 2016 to 2020, the proportion of ART in the total national live births is 1.7%, 1.9%, 2.4%, 2.6% and 2.7%, respectively. There are 540 authorized ART medical institutions and 27 human sperm banks in China, with 540 licensed for AIH, 91 for AID, 415 for conventional IVF and ICSI, and 85 for PGT (Table 1). However, there are great differences in the distribution of ART institutions between the eastern and western regions (Fig. 1A). In the 10 years prior to COVID-19, the total number of ART service cycles in China increased year on year, exceeding 1 million by 2016 (Fig. 1B).

**Table 1:** Statistics on technical categories of art medical services in China

Technical project	Number of institutions
Artificial insemination with husband sperm (AIH)	540
Artificial insemination with donor sperm (AID)	91
Conventional in vitro fertilization and embryo transfer (IVF-ET)	415
Intracytoplasmic sperm injection (ICSI)	
Pre-implantation genetic testing (PGT)	85
Human sperm bank	27

**Fig. 2:**
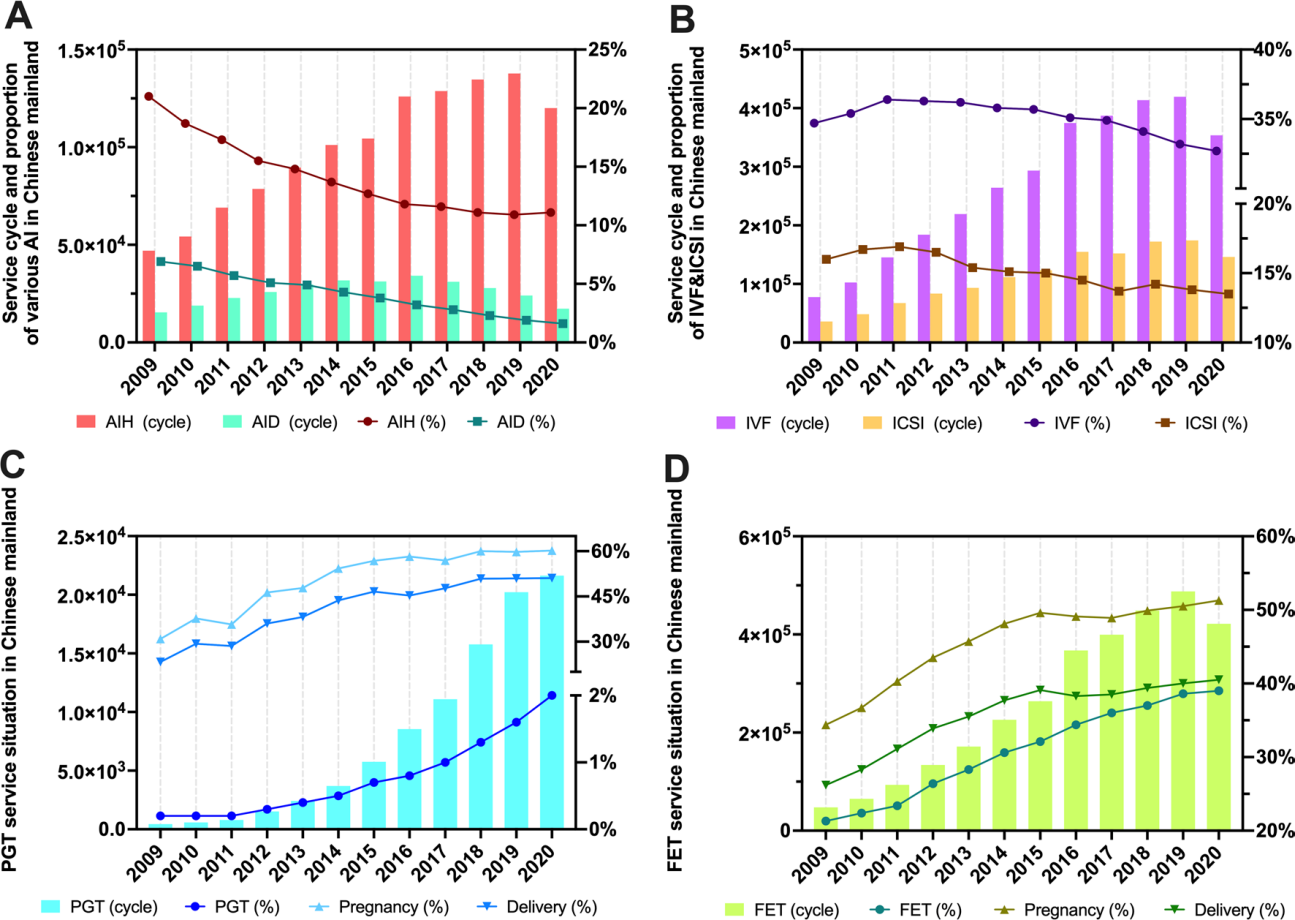
Statistics on the number and proportion of AI, IVF&ICSI, PGT and FET service cycles in China from 2009 to 2020. **a** Service cycle and proportion of various Al in Chinese mainland; **b** Service cycle and proportion of IVF&ICSI in Chinese mainland; **c** PGT service situation in Chinese mainland; **d** FET service situation in Chinese mainland

Given the continuous development of China’s ART, the number of service cycles of various technical categories has shown an upward trend in different degrees, just as the proportion between them has also changed (Table 2). Before COVID-19, the number of AIH service cycles increased each year, but the proportion of AIH and AID have been decreasing year on year (Fig. 2A). Similarly, conventional IVF and ICSI also show the same trend, that is, the number of service cycles have increased while proportions have decreased (Fig. 2B). It is worth noting that the number and proportion of PGT service cycles have increased year on year, especially in the last five years (Fig. 2C). In the 10 years prior to COVID-19, the number and proportion of service cycles of FET steadily increased (Fig. 2D). In addition, clinical pregnancy & delivery rates following PGT and FET showed a steady upward trend (Fig. 2C&D).

**Table 2:** Statistics on the number and proportion of technical service cycles in Chinese mainland from 2009 to 2020

Year	Total	AIH		AID		IVF-ET		ICSI		PGT				FET			
		Cycles	%	Cycles	%	Cycles	%	Cycles	%	Cycles	%	Pregnancy %	Delivery %	Cycles	%	Pregnancy %	Delivery %
2009	223,652	46,967	*21.0*	15,432	*6.9*	77,607	*34.7*	35,784	*16.0*	447	*0.2*	*30.9*	*23.3*	47,638	*21.3*	*34.4*	*26.2*
2010	290,002	54,230	*18.7*	18,850	*6.5*	102,661	*35.4*	48,430	*16.7*	580	*0.2*	*37.7*	*29.3*	64,960	*22.4*	*36.7*	*28.3*
2011	398,925	69,014	*17.3*	22,739	*5.7*	145,209	*36.4*	67,418	*16.9*	798	*0.2*	*35.7*	*28.6*	93,348	*23.4*	*40.3*	*31.1*
2012	507,213	78,618	*15.5*	25,868	*5.1*	184,118	*36.3*	83,690	*16.5*	1,522	*0.3*	*46.3*	*36.0*	133,904	*26.4*	*43.5*	*33.9*
2013	605,441	89,605	*14.8*	29,667	*4.9*	219,170	*36.2*	93,238	*15.4*	2,422	*0.4*	*47.8*	*38.2*	171,340	*28.3*	*45.7*	*35.5*
2014	738,366	101,156	*13.7*	31,750	*4.3*	264,335	*35.8*	111,493	*15.1*	3,692	*0.5*	*54.3*	*43.7*	225,940	*30.6*	*48.1*	*37.7*
2015	822,367	104,441	*12.7*	31,250	*3.8*	293,585	*35.7*	123,355	*15.0*	5,757	*0.7*	*56.8*	*46.6*	263,980	*32.1*	*49.6*	*39.1*
2016	1,067,700	125,989	*11.8*	34,166	*3.2*	374,763	*35.1*	154,817	*14.5*	8,542	*0.8*	*58.2*	*45.3*	367,289	*34.4*	*49.1*	*38.3*
2017	1,109,636	128,718	*11.6*	31,070	*2.8*	387,263	*34.9*	152,020	*13.7*	11,096	*1.0*	*56.9*	*47.7*	399,469	*36.0*	*48.9*	*38.5*
2018	1,212,968	134,639	*11.1*	27,898	*2.3*	413,622	*34.1*	172,241	*14.2*	15,769	*1.3*	*60.0*	*50.9*	448,798	*37.0*	*49.9*	*39.4*
2019	1,263,395	137,710	*10.9*	24,005	*1.9*	419,447	*33.2*	174,349	*13.8*	20,214	*1.6*	*59.8*	*51.0*	487,670	*38.6*	*50.5*	*40.0*
2020	1,081,511	120,048	*11.1*	17,304	*1.6*	353,654	*32.7*	146,004	*13.5*	21,630	*2.0*	*60.2*	*51.1*	421,789	*39.0*	*51.3*	*40.5*

The ratio is indicated in italics

*AIH* Artificial insemination with husband sperm, *AID* Artificial insemination with donor sperm, *IVF-ET* Conventional in vitro fertilization and embryo transfer, *ICSI* Intracytoplasmic sperm injection, *PGT* Pre-implantation genetic testing

**Fig. 3:**
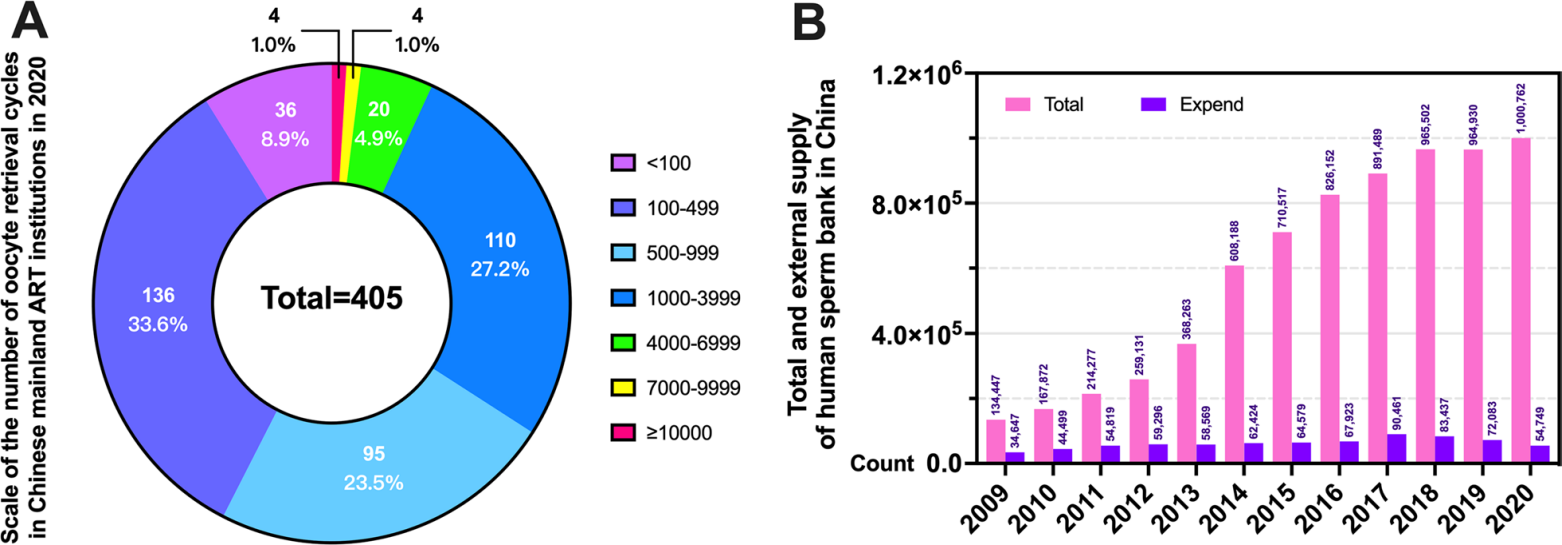
Statistics on the scale of ART institutions and human sperm banks in China. **a** Scale of the number of oocyte retrieval cycles in Chinese mainland ART institutions in 2020; **b** Total and external supply of human sperm bank in China

According to the reported number of oocyte retrieval cycles in 2020, there are 4 super-large ART institutions in China, with 10,000 cycles or more. However, the proportion of institutions with less than 1000 cycles reached 66%, indicating that there are still quite a number of ART institutions in China whose services are not saturated [8] (Fig. 3A). Interestingly, according to published data from China’s human sperm banks, the total number of stocks increased from 134,000 in 2009 to 1 million in 2020. Under the premise of increasing stock, the number of external supply sperm has never exceeded 100 thousand (Fig. 3B). Look at the numbers, it seems that the service ability of Chinese human sperm banks has improved, which can meet the clinical needs of male [9].

But the good news needs further explanation. Chinese regulations limit the number of pregnancies a single donor’s sperm can be used for to 5 women. There are a number of reasons why sperm banks have increased their stock over the past years, firstly based on experiences that numerous cycles using donor sperm are often needed to secure a pregnancy and birth. Secondly, sperm banks anticipated the change in family planning policies which allow women to have up to three children, thereby increasing the number of vials of sperm per donor needed if couples return for a sibling from the same donor. And thirdly, it is possible that sperm banks are hoping that the limit on 5 women’s pregnancies will some day be increased. In short, there are still long waiting lists for donor sperm and sperm banks are still looking to recruit more donors, especially after the pandemic years where potential donors were 'stuck' at home, or as a minimum it was very difficult to donate.

## Impacts of the COVID-19 pandemic on Chinese ART

At present, the dominant strains of the COVID-19 pandemic in the world are Omicron (No.: B.1.1.529) and Delta (No.: B.1.617.2). In China, the subvariants BA.5.2 and BA.7 (abbreviation for BA.5.2.1.7) of BA.5 are more common [10]. Compared to Delta and earlier Alpha, Omicron has a shorter latency (average: 3.42 days, 95% CI: 2.88 ~ 3.96 days) [11] and shorter symptom duration (average: 6.87 days, 95% CI: 6.58 ~ 7.16 days) [12]. Omicron is also characterized by multiple mutations and is highly infectious [13].

According to China’s National Expert Group on Quality Management of Assisted Reproductive Technology, 63.6% of ART providers and 95.5% of human sperm banks suspended operations due to COVID-19 pandemic [14]. When comparing the service volumes of various types of ART and sperm banks in China from January to April in 2019 and 2020, we can see that the total number of outpatients, AIH & AID cycles, aspiration cycles, fresh embryo transfer cycles, and FET cycles were 55.0%, 53.5%, 47.2%, 50.8%, 47.7% and 62.9%, respectively, making the average recovery rate of ART service volume 52.9%. The total number of sperm donors, the number of straws of qualified semen, the total number of straws of donor semen, the total number of self-sperm preservations, and the number of straws of self-sperm preservations were 39.3%, 26.2%, 39.9%, 48.9% and 46.7%, respectively, amounting to an average recovery rate of sperm bank service volume of 61.8% (Table 3).

**Table 3:** Comparison of business service volumes of various ART and sperm banks in China from January to April in 2019 and 2020

	Total services Jan.–Apr		Recovery ratio (%)				
	2020	2019	Jan.–Apr	Jan	Feb	Mar	Apr
ART service volumes							
Outpatients	4,952,295	9,009,000	*55.0*	*69.7*	*27.8*	*48.7*	*65.1*
AIH	21,229	39,711	*53.5*	*68.9*	*19.3*	*41.8*	*67.0*
AID	3,899	8,258	*47.2*	*59.2*	*16.1*	*37.1*	*61.2*
Aspiration cycles	86,397	170,161	*50.8*	*65.3*	*45.8*	*32.5*	*57.7*
Fresh embryo transfer cycles	31,866	66,745	*47.7*	*65.3*	*42.2*	*29.8*	*50.8*
Frozen embryo transfer cycles	90,550	143,854	*62.9*	*75.3*	*44.7*	*41.4*	*82.6*
Average	864,372.7	1,572,954.8	*52.9*	*67.3*	*32.7*	*38.6*	*64.1*
Sperm banks service volumes							
Sperm donors	2,967	7,544	*39.3*	*53.0*	*2.2*	*14.6*	*71.5*
Tubes of qualified semen	8,741	33,409	*26.2*	*52.5*	*1.5*	*7.5*	*31.0*
Tubes of external donor semen	7,219	18,107	*39.9*	*62.4*	*6.3*	*15.5*	*65.5*
Self-sperm preservation	911	1,864	*48.9*	*64.6*	*11.8*	*38.6*	*74.5*
Tubes of self-sperm preservation	2,992	6,410	*46.7*	*61.5*	*13.5*	*37.6*	*66.5*
Average	4,566.0	13,466.8	*40.2*	*58.8*	*7.1*	*22.8*	*61.8*

The ratio is indicated in italics

Status of institutional responses: among all surveyed institutions, there were 475 ART centers (91.9%) and 22 sperm banks (81.5%) that completed the online questionnaire

In addition, data from Beijing ART Quality Control Center shows that, from January to April 2020, the total outpatient volume of 12 ART centers in Beijing decreased by 54.4%, compared with the same period in 2019. Fresh embryo transfers and thawed embryo transfers decreased by 78.7% and 51.5%, respectively. Normal ICSI fertilization rates and clinical pregnancy rates of fresh & thawed embryo transfers decreased significantly, while the percentage of cycles without transferable embryos increased significantly [15]. Similarly, data from the Sichuan Academy of Medical Sciences show that the number of outpatients in January–Mar. and April–June 2020 decreased by 53. 22% and 18. 97%, respectively, compared with the same period in the previous year. And the number of service cycles decreased by 69. 31% and 30. 98%, respectively. Thankfully, the number of outpatients in April–June 2020 increased by 79. 19% compared with January–March In the same period of 2019, the number of outpatients increased by only 3. 46%, indicating that ART medical services have been significantly restored [16].

**Fig. 4:**
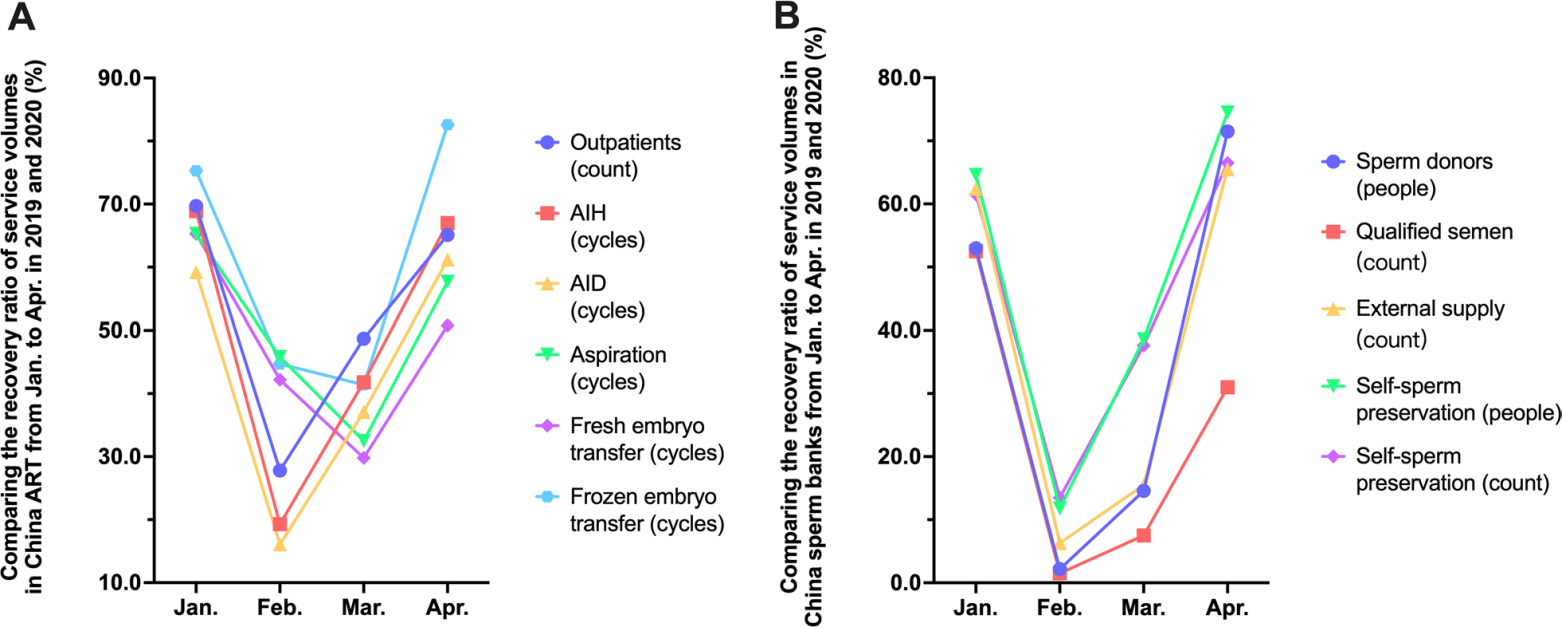
Statistics on the number of oocyte retrieval cycles in China. **a** Comparing the recovery ratio of service volumes in China ART from Jan. to Apr. in 2019 and 2020 (%); **b** Comparing the recovery ratio of service volumes in China sperm banks from Jan. to Apr. in 2019 and 2020 (%)

In terms of months, the total number of outpatients, AIH & AID cycles were the lowest in February, when the domestic epidemic was most severe, and recovered rapidly thereafter; the recovery rates of egg retrieval, fresh transplantation and thawing transplantation cycles were the lowest in March due to a certain "delay effect" in the downward trend (Fig. 4A). The recovery rate of all types of sperm bank services was the lowest in February, when the epidemic was most severe, and recovered rapidly thereafter (Fig. 4B). As of the end of May 2020, China, as an early country affected by this outbreak achieved a national resumption rate of ART medical services of 99.2% and that of human sperm banks of 100.0%, with a total of 8 confirmed cases of new crown infection, including 5 cases of infection among ART medical staff and 3 cases among patients attending the clinic, and no cases of infection among medical staff in sperm banks [14]. There is no doubt that China’s prevention and control initiatives for this outbreak have been successful.

The human sperm bank of Hunan province (hospital of CITIC-Xiangya), the first and largest in China, analyzed the recruitment of human sperm bank in the post-epidemic period [17]. This study showed that qualification, semen concentration and sperm viability rates at primary screening have significantly decreased in the post-epidemic period. Moreover, there are significant differences in age, abstinence days and BMI among the primary screening sperm donors before and after COVID-19 (Table 4). At the same time, in the three groups, 20 ~ 24, 25 ~ 29 and 30 ~ 34 years old, the qualification rates at primary screening were significantly lower than those before COVID-19 (Table 5). In addition, the occupation, age and education of sperm donors have no significant influence on the qualification rate of sperm donors in the post-epidemic period, but there are significant differences in the season of sperm donation (Table 6). The results showed that the epidemic situation had a great impact on the recruitment of sperm donors in human sperm banks. At the same time, study authors suggest that the publicity of sperm donor recruitment should be strengthened and the mental health of sperm donors should be supported.

**Table 4:** Comparison of semen quality and baseline data of primary screening donors before and after COVID-19

	Before	After	*p *value
Qualified rate	27.04 (1090/4031)	19.68 (935/4752)	0.000
Semen concentration	50 (30, 62)	48 (30, 64)	0.001
Sperm viability	50 (43, 50)	50 (40, 50)	0.000
Age	21 (20, 24)	21 (20, 23)	0.000
Abstinence days	5 (4, 7)	4 (4, 5)	0.000
BMI	21.3 (19.6, 23.4)	21.5 (19.8, 23.7)	0.001

**Table 5:** Comparison of qualified rate of donors in different age groups before and after COVID-19

Group (years old)	Before	After	*p *value
20–24	26.66	19.95	0.000
25–29	30.78	18.98	0.000
30–34	26.05	16.79	0.007
≥ 35	10.34	27.27	0.319

**Table 6:** Statistics of distribution characteristics of sperm donors after COVID-19

	Total	Qualified number	Qualified rate (%)	*p*-value
Occupation				
Student	2857	572	20.02	*0.463*
Social personnel	1895	363	19.16	
Age				
20–24	3919	782	19.95	*0.530*
25–29	548	104	18.98	
30–34	274	46	16.79	
≥ 35	11	3	27.27	
Education				
Graduate degree	88	13	14.77	*0.132*
Bachelor's degree	1477	318	21.53	
College degree	2745	521	18.98	
High school diploma	442	83	18.78	
Season of sperm donation				
Mar.–May	1380	314	22.75	*0.000*
June–Aug	1233	235	19.06	
Sep.–Nov	1306	264	20.21	
Dec.–Feb	833	122	14.65	

The *p*-value is indicated in italics

## Infection prevention and control at ART institutions

COVID-19 continues to spread around the world, and based on the long-term, complex and recurrent nature of this pandemic, prevention and control efforts in various countries have been normalized. Moreover, preparedness for future infectious disease outbreaks including how to strengthen the construction and management of safety protocols in the delivery of medical services have received wide attention. The completion of ART services involves the processes of ovarian/testicular tissue acquisition, gamete acquisition, in vitro fertilization, embryo culture, embryo testing, embryo implantation, etc., and requires the coordination of multiple components, such as outpatient departments, inpatient departments, operation rooms, laboratories, and sperm banks. To address the particular characteristics of ART services work, the Chinese Expert Group on Quality Management of Assisted Reproductive Technology has developed a corresponding expert consensus on prevention and control measures as a part of normalized management protocols [18]–[20].

**Fig. 5:**
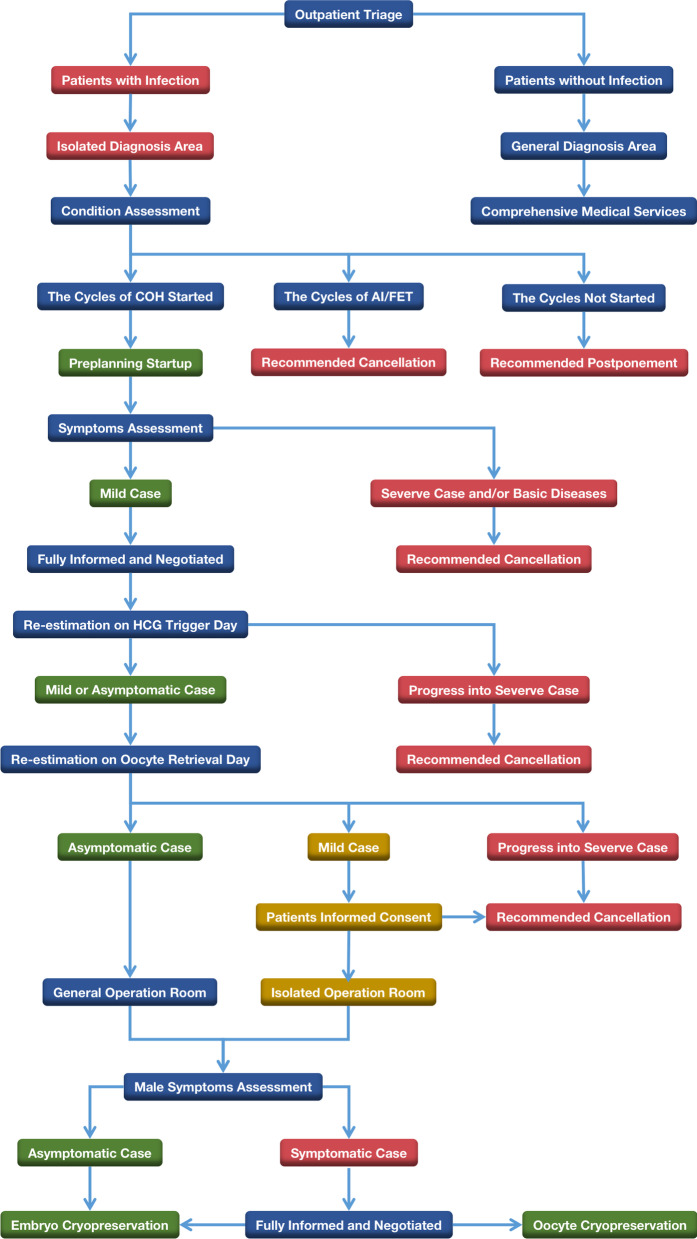
Recommendations from Chinese experts for the out-patient diagnosis and treatment process in ART institutions during the pandemic of COVID-19

Based on service characteristics and clinical experience, Chinese experts mapped out outpatient diagnosis and treatment processes for ART medical institutions [19] (Fig. 5). Considering the characteristics of ART services work, outpatient clinics should realize online appointments, consultations with specific times and areas, and dynamic arrangement of consultation areas [21]. During a pandemic, management measures such as limiting hospitalization, speeding up discharge, reducing the number of patients in hospital, increasing space and orderly visiting should be implemented on inpatient wards [22]. Specifically, continuous body temperature monitoring of patients and encouragement of family members to use online methods for communication are needed, and family visits to the ward are not recommended [23].

The operating room, as a pivotal clinical platform, is a high-risk area for cross-infection. Chinese scholars have developed a safety management model known as "one completeness”, “two verifications” and “three eliminations " in reproductive operating rooms, which has achieved good results of "four zeros" and is worth promoting [24, 25]. The term "one completeness " refers to the complete laboratory data provided by the patient before surgery. The "two verifications" refers to the verifications of identity information and epidemiological investigation data on the day of the patient’s surgery. The "three eliminations" include routine disinfection for conservators; upgraded disinfection measures for special conservators; and elimination of anxiety and fear of conservators. The final objective is "four zeros", i.e., zero errors in identity verification, zero COVID-19 patients missed, zero in-hospital cross-infection, and zero complaints.

**Fig. 6:**
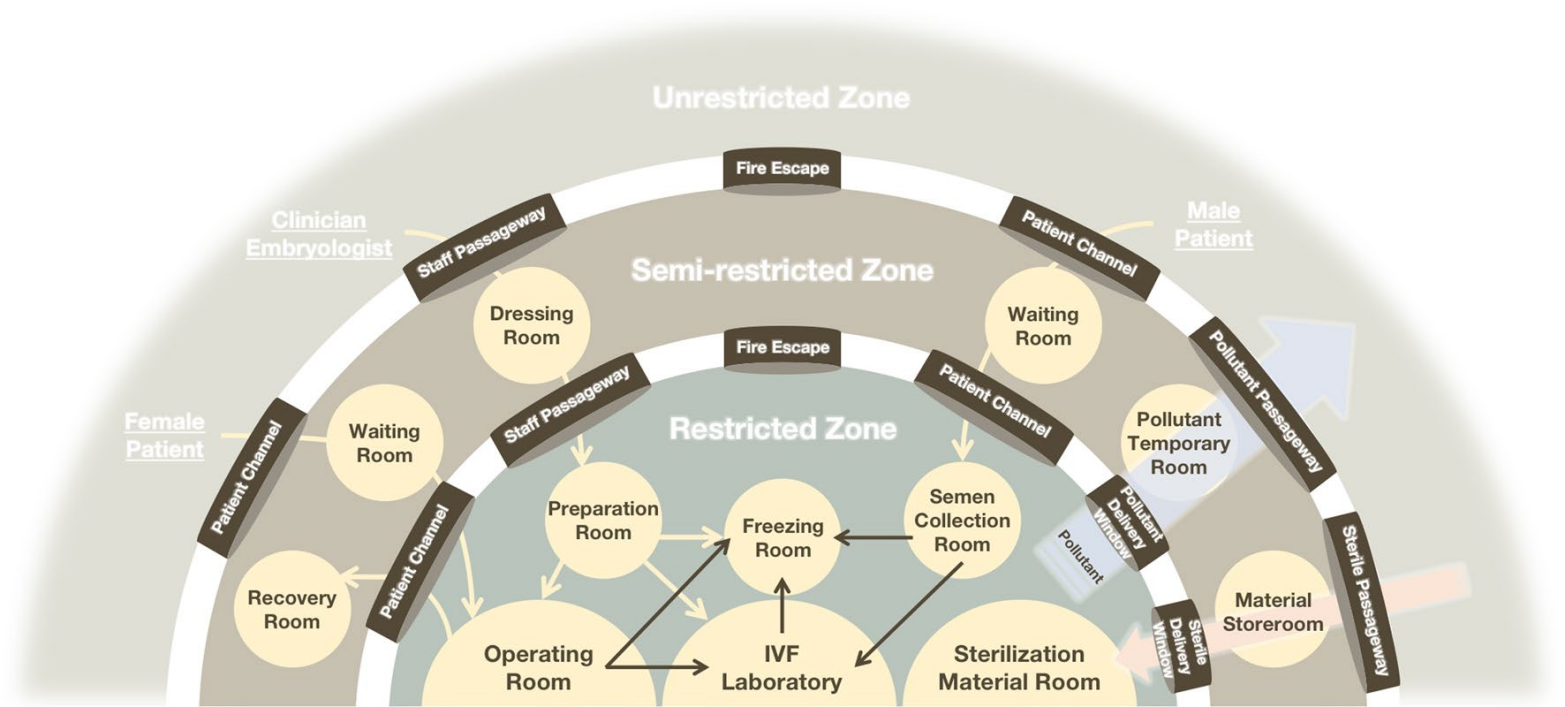
Pattern diagram of "three zones and five channels" biosafety protection system in IVF laboratory. "Three zones" including restricted (clean) zones, semi-restricted (semi-contaminated) zones, and unrestricted (contaminated) zones; “Five channels” including medical staff channel, patient channel, pollutant channel, sterilization material channel, and fire escape channel

Laboratories occupy a very important place in carrying out ART services, directly carrying the processes of manipulating sperm, eggs and embryos. However, the relatively confined environment is more prone to cross-infection, and thus strengthening biosafety protection is particularly important. Chinese scholars have summarized countermeasures against the risk of viral contamination in reproductive laboratories, and suggested the establishment of a biosafety protection system of "three zones and five channels" (Fig. 6), which is divided into unrestricted (contaminated), semi-restricted (semi-contaminated), and restricted (clean) zones, with double channels without crossover and different levels of decontamination. Buffer rooms are provided between zones for hand washing, shoe changing and other protective operations [26].

Since human activities with infectious diseases can produce viral aerosols, they will inevitably be deposited on the bodies and hands (other hand-accessible surfaces) of people around them. In addition to clothing, ventilation ducts, and material packaging, the ART services process should pay more attention to the prevention and control of potential specialist transmission routes caused by male semen and female follicular fluid. According to existing research reports, SARS-CoV-2 was not detected in human follicular fluid of infected females [27], while the virus has been detected in the semen of a few male patients at the first stage of infection [28]. We do not fully understand SARS-CoV-2, and although there is no evidence of adverse effects of SARS-CoV-2 on gametes and embryos, there is a potential for transmission through germ cells themselves, virus particles falling into culture micro droplets, and cross-infection in liquid nitrogen [29, 30]. Therefore, the potential for contamination or infection should be taken seriously.

## Prevention and control measures in human sperm banks

Currently, human sperm banks are mainly involved in four major services of collection, testing, preservation and provision of sperm. Because of the uncertainty of the effect of SARS-CoV-2 on sperm and the potential cross-infection of the virus in liquid nitrogen, Chinese experts emphasized that the prevention and control measures for sperm banks under the epidemic norm should be improved to provide safe and convenient services for sperm donors and autologous sperm preservation patients. The Chinese expert consensus statement also provides meticulous arrangements for prevention and control in the management of sperm banks [18, 31].

Sperm banks should start an online booking platform, make appointments in batches at different times to donate sperm or store sperm, and to fully ensure safe distance.The number of people received at the same time should not exceed 1/3 of the maximum capacity of the reception room; people confirmed as infected, feverish, suspected of infection and people with a history of close contact should not be allowed to enter the sperm bank. Sperm donors or autologous sperm preservation patients are required to check their body temperature and complete an epidemiological investigation for each sperm retrieval. To strengthen the management of semen freezing during the special period fully enclosed freezing carriers and vapor-phase liquid nitrogen tanks are recommended for storage of semen specimens to reduce the risk to semen users. For all sperm donors and autologous sperm preservation patients received during an epidemic, frozen semen from each donor or autologous sperm preservation patient should be stored in emergency vapor-phase liquid nitrogen tanks, with a moderate physical distance between each stored specimen. After 14 days, depending on the follow-up results and opinions of the reception department, they should either be placed in a conventional tank or destroyed. Positive specimens of autologous sperm preservation patients that are not suitable for destruction must be stored in separate liquid nitrogen tanks and properly marked and recorded.

## Special reproductive care in the post-pandemic era

SARS-CoV-2 and earlier SARS-COV (severe acute respiratory syndrome coronavirus) had nearly 80% homologous sequence of amino acid that could both invade host cells via ACE2 (angiotensin converting enzyme 2) and TMPRSS2 (cellular serine protease) [32]. A large number of studies have found that the ACE2 is abundantly expressed in the human reproductive system [33, 34], and thus the reproductive function is more vulnerable to SARS-CoV-2. Based on current research reports, there is no clear evidence yet that SARS-CoV-2 infection has a negative impact on ovarian function and IVF outcomes [35, 36]. But, there is some evidence that the testes are a higher risk organ for infection, which has a molecular basis for binding to SARS-CoV-2 [37]–[39]. Based on the above, Chinese scholars highlight that attention should be paid to young patients with fertility requirements during and after COVID-19, and emphasize the importance of fertility evaluation and clinical intervention [40].

In order to block the spread of SARS-CoV-2, disinfectants of various types and forms are massively, widely and frequently used in medical & health institutions, families & communities, public places and so on. Previous studies have shown that hypochlorite can significantly increase the sperm malformation rates of mice by gavage [41]. The mortality of zebrafish embryos exposed to peracetic acid (0.75 mg/L, 2h) is as high as 89.6% ± 3.4% [42]. A large number of animal experiments have proved that phenolic disinfectants can cause abnormality of fetal development and spontaneous abortion [43, 44]. In addition, the effective component of the iodine-containing disinfectant is iodine, while the sperm concentration will decrease after excessive iodine intake [45]. Couples should be reminded that during ART treatment, 75% alcohol and chlorine-containing disinfectants within safe concentration are recommended. Excessive use of disinfectants should be discouraged, as it may cause damage to the reproductive system, gametes and zygotes. Large-scale outbreak of emerging infectious diseases may cause panic among medical workers, patients and the public, inducing depression, anxiety, posttraumatic stress disorder (PTSD) and so on [46–51]. The irrational tension or fear by above stressor stimulation activate the stress response of the central nervous system, and thus affect reproductive function [52–54]. Previous studies have shown that stress and negative emotions (such as depression and anxiety) can affect various sperm parameters, including semen volume, sperm concentration, sperm motility, DNA fragmentation and so on [55–58]. Female reproductive systems are also affected by mental and psychological factors. Studies have found that stress can cause the accumulation of reactive oxygen species (ROS) in the ovary and is related to spontaneous abortion [59, 60]. Therefore, reliable information and psychological counseling services should be provided to couples with fertility requirements during a pandemic. Timely and reasonable guidance in the face of negative emotions from stress responses is needed to avoid irrational fear and excessive stress.

ART treatments are often long processes, so patients may be infected at different stages of treatment. In principle, all ART activities should be suspended during infection. In addition, there are no large data to demonstrate a significant adverse effect of vaccination on ART outcomes. It is recommended that the analysis of seminal parameters should be re-examined 2 months after SARS-CoV-2 vaccination, and that ART treatments can proceed if there are no abnormalities [61, 62]. For patients who have commenced treatment, cancelling treatment requires comprehensive consideration of the possible physical & psychological damages, economic loss and other factors.

## Conclusion

In recent years, new and sudden outbreaks of infectious diseases all over the world have become more frequent, such as ongoing COVID-19, mpox (formerly monkeypox), influenza A (A/PR/8/34 strain, H1N1), Ebola hemorrhagic fever and cholera outbreaks. It is especially important that the ART institutions and human sperm banks pay attention to improving their infection prevention and control capabilities. The decisions and measures adopted in China as a consequence of the COVID-19 pandemic can make a valuable contribution to the global ART community. With the aim of making progress together with other countries in the world, Chinese scholars have proactively tackled the key issues concerning safe ART development during the pandemic and are keen to share China’s experiences and solutions.

## Data Availability

The datasets used or analyzed during the current study are available from the corresponding author on reasonable request.

## Abbreviations

AID: Artificial insemination with donor sperm
AIH: Artificial insemination with husband sperm
ART: Assisted Reproductive Technology
ASRM: American Society for Reproductive Medicine
BFS/ARCS: British Fertility Society/Association of Reproductive and Clinical Scientists
BMI: Body mass index
CFAS: Canadian Fertility and Andrology Society
COVID-19: Corona Virus Disease 2019
CSRM: Chinese Society of Reproductive Medicine
ESHRE: European Society of Human Reproduction and Embryology
FET: Frozen embryo transfer
H1N1: Hemagglutinin 1 neuraminidase 1
ICSI: Intracytoplasmic sperm injection
IVF: Conventional in vitro fertilization
PGT: Pre-implantation genetic testing
SARS: Severe acuterespiratory syndrome
SARS-CoV-2: Severe acute respiratory syndrome coronavirus 2
